# Clinical and laboratory findings and PCR results in severe and non-severe COVID19 patients based on CURB-65 and WHO severity indices

**DOI:** 10.1186/s12985-021-01658-1

**Published:** 2021-09-17

**Authors:** Farnaz Karimi, Mahshid Saleh, Amir Abbas Vaezi, Mostafa Qorbani, Foroogh Alborzi Avanaki

**Affiliations:** 1 Department of Pathology, Alborz University of Medical Sciences, Karaj, Iran; 2 Department of Applied Cell Sciences, Tehran University of Medical Sciences, Tehran, Iran; 3 Department of Internal Medicine, Alborz University of Medical Sciences, Karaj, Iran; 4 Non-Communicable Diseases Research Center, Alborz University of Medical Sciences, Karaj, Iran; 5 Chronic Diseases Research Center, Tehran University of Medical Sciences Endocrinology and Metabolism Research Institute, Tehran, Iran; 6 Department of Gastroenterology, Imam Khomeini Hospital, Tehran University of Medical Sciences, Tehran, Iran

**Keywords:** COVID-19, rRT-PCR, Clinical and laboratory findings, CURB-65, Severe, Non-severe

## Abstract

**Background:**

The importance of clinicolaboratory characteristics of COVID-19 made us report our findings in the Alborz province according to the latest National Guideline for the diagnosis and treatment of COVID-19 in outpatients and inpatients (trial five versions, 25 March 2020) of Iran by emphasizing rRT-PCR results, clinical features, comorbidities, and other laboratory findings in patients according to the severity of the disease.

**Methods:**

In this study, 202 patients were included, primarily of whom 164 had fulfilled the inclusion criteria. This cross-sectional, two-center study that involved 164 symptomatic adults hospitalized with the diagnosis of COVID-19 between March 5 and April 5, 2020, was performed to analyze the frequency of rRT-PCR results, distribution of comorbidities, and initial clinicolaboratory data in severe and non-severe cases, comparing the compatibility of two methods for categorizing the severity of the disease.

**Results:**

According to our findings, 111 patients were rRT-PCR positive (67.6%), and 53 were rRT-PCR negative (32.4%), indicating no significant difference between severity groups that were not related to the date of symptoms' onset before admission.

Based on the National Guideline, among vital signs and symptoms, mean oxygen saturation and frequency of nausea showed a significant difference between the two groups (*P* < 0.05); however, no significant difference was observed in comorbidities. In CURB-65 groups, among vital signs and comorbidities, mean oxygen saturation, diabetes, hypertension (HTN), hyperlipidemia, chronic heart disease (CHD), and asthma showed a significant difference between the two groups (*P* < 0.05), but no significant difference was seen in symptoms.

**Conclusion:**

In this study, rRT-PCR results of hospitalized patients with COVID-19 were not related to severity categories. From initial clinical characteristics, decreased oxygen saturation appears to be a more common abnormality in severe and non-severe categories. National Guideline indices seem to be more comprehensive to categorize patients in severity groups than CURB-65, and there was compatibility just in non-severe groups of National Guideline and CURB-65 categories.

## Introduction

In December 2019, Hubei province in China turned into the epicenter for the spread of pneumonia with an incognito etiology. January 7, 2020, Chinese researchers had detected a new coronavirus, namely severe acute respiratory syndrome coronavirus 2 (SARS-CoV-2; formerly known as 2019-nCoV) from the patients infected with pneumonia [1, 2], which was named coronavirus disease 2019 (COVID-19) in 2020 by WHO. In Iran, the first cases of COVID-19 were officially announced between February 19 and 23, 2020. It soon became apparent that Iran is one of the countries worst hit by the COVID-19 outbreak [3].

Coronaviruses are enveloped non-segmented positive-sense RNA viruses belonging to the family Coronaviridae, broadly distributed in humans and other mammals [4].

The clinical perspective of SARS-CoV-2 infection appears to be broad, containing asymptomatic infection, mild upper respiratory tract disease, severe viral pneumonia with respiratory failure, and even decease and a large number of hospitalized patients with pneumonia in Wuhan [5–7].

According to the latest National Guideline for the diagnosis and treatment of COVID-19 in outpatients and inpatients (trial five versions), the definite diagnosis of COVID-19 must be confirmed by reverse transcription-polymerase chain reaction (rRT-PCR). For probable cases considered patients, radiological findings such as ground-glass opacities, multifocal patchy consolidation, and interstitial changes with a peripheral distribution in a chest CT scan are strongly recommended [8]. However, with the limitations of sample collection, transportation, and kit performance, rRT-PCR's total positive rate for throat swab samples was reported to be 30–60% at initial presentation [9]. rRT-PCR’s low sensitivity implies that many COVID-19 patients may not be identified and could not receive appropriate treatment promptly in the current emergency status. As a standard imaging tool for pneumonia diagnosis, chest CT is relatively easy to perform and can quickly diagnose the disease [10].

The typical features have also been observed in patients with negative rRT-PCR results in having clinical symptoms. It has been noted in small-scale studies that the current rRT-PCR testing has limited sensitivity, while chest CT can reveal pulmonary abnormalities consistent with COVID-19 in patients with initially negative rRT-PCR results [11, 12]. In an epidemic area, negative rRT-PCR but positive CT features can still be highly suggestive of COVID-19, which has important clinical and societal implications. Rapid detection with high sensitivity of viral infection may allow for better control of viral spread. A second limitation is that there is little clinical and laboratory data during this urgent period when regional hospitals are overloaded [13].

Due to limited access to the rRT-PCR test at the early weeks of the COVID-19 pandemic, many probable cases were hospitalized in Alborz province of Iran in two academic hospitals according to CT findings, vitals sign, and initial laboratory tests such as CRP and lymphocyte counts and their management started immediately.

This study's first purpose was to better understand the relationship between real-time reverse transcription-polymerase chain reaction (rRT-PCR) on throat swab samples, particularly concerning patients' severity status and comorbidities. Other aims were to review the initial clinical characteristics and comorbidities of hospitalized patients with clinic radiological findings suggestive of COVID-19 referred to our centers and compare those findings between two severity groups. The first classification was based on severity indices provided by National Guideline [14], and the second was CURB-65 classification primarily used for bacterial pneumonia, and it was attempted to find if there is any compatibility between the two severity classifications.

## Methods

### Study design and setting

**Fig. 1:**
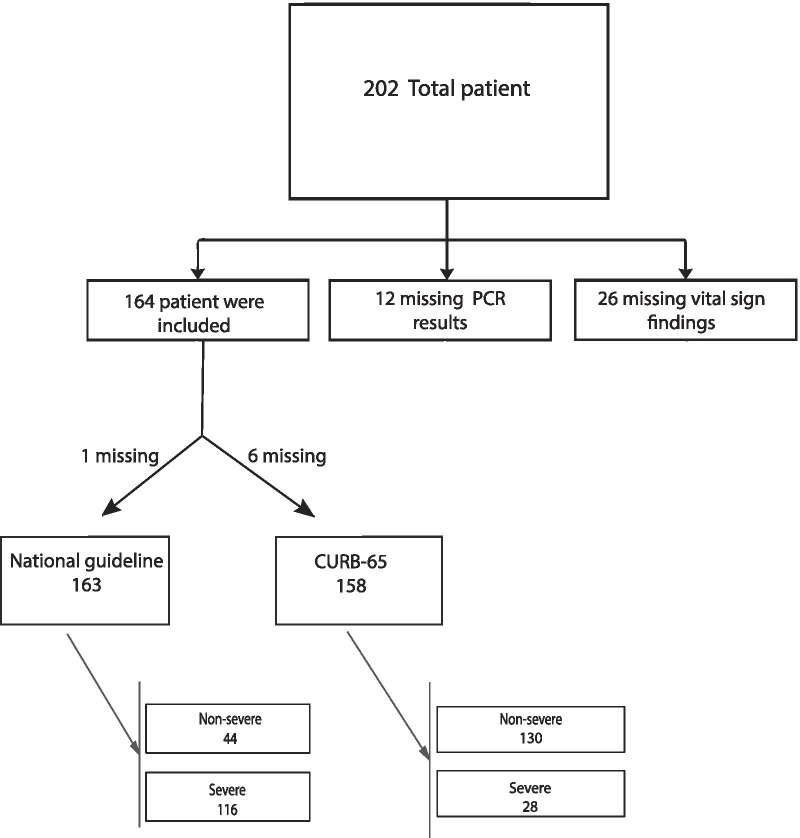
Flowchart of patients selection and study path

This cross-sectional study was conducted between March 5 and April 5, 2020, at two referral hospitals in the Alborz province of Iran that provided care for COVID-19 patients. In this study, 202 patients were included, primarily of whom 164 had fulfilled the inclusion criteria (Fig. 1). The ethics committee approved this study of Alborz University of Medical Sciences (ABZUMS) (IR.ABZUMS.REC 1398.267). Written informed consent was taken for data collection from all patients, and data were collected from patient's medical records and patients' self-reports of severe or non-severe disease.

Probable cases who were admitted to the hospital according to National Guideline [8] were tested for rRT-PCR of oropharyngeal swab specimens by well-trained constant operators and sample collectors.

### Variables

The collected data included patients' demographic information, comorbidities, date of symptoms onset before admission, triage vitals, O2 saturation, initial laboratory tests (complete blood counts, biochemistry parameters, inflammatory indices), and initial lung CT scan results during the hospitalization period.

Initial laboratory testing was performed as the first test results available typically within 24 h of admission. Twenty-six patients had missing data of initial vitals, and 12 patients had no rRT-PCR results.

The COVID-19 symptoms included nonproductive cough, fever, chill, dyspnea, sore throat, headache, dizziness, weakness, muscular pain, diarrhea, abdominal pain, anorexia, nausea, vomiting, and the comorbidities were diabetes, hyperlipidemia, hypertension, chronic heart disease (CHD), chronic kidney disease (CKD), asthma, chronic obstructive pulmonary disease (COPD), cirrhosis, autoimmune disease, history of malignancy, recent chemoradiotherapy, current steroid, and immunosuppressant drug use. Triage vitals such as temperature (Temp), pulse rate (PR), respiratory rate (RR), systolic and diastolic blood pressure (SBP-DBP), oxygen saturation (Sat O2) were also considered.

Two severity criteria were used to classify patients in severe and non-severe groups: CURB-65 and National Guideline Criteria [14].

### rRT-PCR

Viral RNAs were extracted from samples using Qiagen Viral Nucleic Acid Kit (QIAcub HT), and a reverse transcription-polymerase chain reaction (rRT-PCR) was performed (Molbiol, Germany) using an approved commercial kit specific for 2019-nCoV detection. Cycling conditions for amplification of E and RdRP genes were 50 °C for 30 min, 95 °C for 2 min, followed by 45 cycles of 95 °C for 10 s and 60 °C for 30 s. A cycle threshold value of < 36 Ct was defined as a positive test result.

### Definition

1. A confirmed case was defined as a suspected one with the laboratory test for COVID-19 from the respiratory specimens showing positive rRT-PCR assay results.
2. A suspected case was defined as a case that fulfilled the following criteria: fever, radiographic evidence of pneumonia, low or normal WBC count or low lymphocyte counts in the clinic, admission to the ward due to one or more of these reasons: 1) Clinical signs and symptoms, 2) Lung infiltration in CT Scan, 3) O2 Saturation < 93, 4) Respiratory rate > 30.
3. A severe case was defined according to National Guideline [10] as the presence of at least one of the following criteria: Respiratory Rate > 24, Heart Rate > 125, O2 Saturation < 90% on ambient air; Vital sign and CRP > 100, LDH > 245u/l among laboratory data.
4. CURB-65 is a clinical prediction rule that has been validated for predicting mortality in community-acquired pneumonia. The score is an acronym for each of the risk factors measured, which has one point for a maximum score of 5:
  Confusion of new-onset
  Blood Urea nitrogen greater than 19 mg/dL (7 mmol/l)
  Respiratory rate of 30 breaths per minute or greater
  Blood pressure <90 mmHg systolic or diastolic blood pressure ≤60 mmHg
  Age 65 or older.

Having each of these items is considered one score for the patient. In total, between 0 and 5 scores are given to each patient [15, 16].

### Statistical analysis

The Kolmogorov–Smirnov test for normality evaluated continuous variables. Typical data and non-Gaussian distributed data were expressed as mean ± standard deviation (SD), and the median and interquartile range (IQR), respectively, compared using t-test and Mann–Whitney U test between severity groups. Categorical variables were presented as frequency and percentage. Chi-square test and cross-tabulation were employed for testing the relationships between categorical variables in severity groups. All statistical analyses were performed with SPSS software version 22.0, and *P* value < 0.05 was considered statistically significant.

## Results

Between March 5 and April 5, 2020, hospitalized patients with SARS-CoV-2 symptoms were diagnosed at two medical centers of Alborz province of Iran. A total of 164 patients were included, mainly male (56.1%, 43.9% female), and a mean age of 54 years (Table 1).

**Table 1:** Clinical characteristics of patients infected with SARS-CoV-2

Variables	Total (n = 164)
Gender. no (%)	
Male	92 (56.1)
Female	72 (43.9)
Age	54.0 (15.1)
Initial vital sign mean (SD)	
RR breaths/min	19.2 (2.8)
Temp	37.0 (0.9)
PR	99.1 (19.2)
SBP	130.2 (20.1)
DBP	79.3 (12.7)
O2 SAT	91.0 (6.5)
Symptoms. no (%)	
Cough	118 (71.8)
Fever	83 (50.9)
Chill	60 (36.8)
Dyspnea	93 (57.1)
Sore throat	30 (18.4)
Headache	51 (31.3)
Dizziness	37 (22.7)
Weakness	80 (49.1)
Muscular pain	75 (46.0)
Diarrhea	27 (16.6)
Abdominal pain	14 (8.6)
Anorexia	73 (44.8)
Nausea	52 (31.9)
Vomiting	30 (18.4)
Comorbidity no (%)	
Diabetes	38 (23.3)
Hyperlipidemia	10 (6.1)
Hypertension	38 (23.3)
Chronic heart disease	24 (14.7)
Chronic kidney disease	1 (0.6)
Asthma	11 (6.7)
COPD	0 (0.0)
Cirrhosis	0 (0.0)
Autoimmune disease	1 (0.6)
History of malignancy	1 (0.6)
Recent chemoradiotherapy	1 (0.6)
Current steroid use	1 (0.6)
Immunosuppressant drug use	1 (0.6)

*RR* respiratory rate, *Temp* temperature, *PR* pulse rate, *Sys BP* systolic blood pressure, *Dias BP* diastolic blood pressure, *O2 Sat* O2 saturation, *CHD* chronic heart disease, *CKD* chronic kidney disease, *COPD* chronic obstructive pulmonary disease

All the patients had similar chest CT scan findings reported by the same radiologist on the admission day or before. Oropharyngeal sampling for COVID-19 rRT-PCR was done on admission for all the patients using the same protocol performed by well-trained staff and rRT-PCR device operators. In our study, 111 patients were rRT-PCR positive (67.6%), and 53 were rRT-PCR negative (32.4%) [17].

Clinical features are summarized in (Table 1). In total, nonproductive cough 118 (71.8%), dyspnea 93 (57.1%), and fever 83 (50.9%) were the most common symptoms. Other symptoms were chills, sore throat, headache, dizziness, weakness, muscular pain, diarrhea, abdominal pain, anorexia, nausea, vomiting—no patients presented with severe acute respiratory distress syndrome.

Vital signs were also extracted on admission, including mean heart rate 99.1 (19.2), mean respiratory rate 19.2 (2.8), mean systolic blood pressure 130.2 (20.1) and mean diastolic blood pressure 79.3 (12.7), mean body temperature 37 (0.9) as well as mean O2 saturation on admission day 91 (6.5) (Table 1).

The frequency of tachypnea with RR > 24 on the admission day was 9 (5.5%), Temp > 37.8 was 38 (23%), HR > 125 (10.3%) and SO2 < 90 (22.4%). Fully conscious patients were 161 (98.8%), and two patients were in stupor condition (1.2%). The median time from disease onset to admission was 6.5 days (IQR 4–8.7) (Tables 1 and 2).

**Table 2:** Frequency of specific clinical and laboratory findings of patients infected with SARS-CoV-2, total

Specific variables and lab findings n/N (%)	Total
RR > 24	9/163 (5.5)
Temp > 37.8	38/163 (23.3)
PR > 125	17/163 (10.4)
So2 < 90%	37/163 (22.7)
Fully conscious patients	161/163 (98.8)
Non fully conscious patients	2/163 (1.2)
CRP > 6	128/159 (80.5)
CRP > 100	15/150 (9.4)
PCR positive	111/163 (68.1)
PCR negative	52 (31.9)
Lymph < 1100	67/162 (41.4)
LDH > 245	98/104 (94.2)

*RR* respiratory rate, *Temp* temperature, *PR* pulse rate, *O2 Sat* O2 Saturation, *CRP* C reactive protein, *PCR* polymerase chain reaction, *Lymph* lymphocyte count, *LDH* lactate dehydrogenase

^*^This data is Median (IQR)

Among the history of comorbidities, diabetes was the most common with 38 (23.3%), the rest of comorbidities were hyperlipidemia 10 (6.1%), hypertension 38 (23.3%), chronic heart disease 24 (14.7%), chronic kidney disease, and pulmonary disease 11 (6.7%). Other comorbidities in Table 1 were cirrhosis, autoimmune disease, history of malignancy, recent chemoradiotherapy, current steroid use, immunosuppressive drug use (Table 1).

The cellular count and biochemical parameters obtained on the first day of admission are listed in Table 3.

**Table 3:** Initial laboratory findings of patients infected with SARS-CoV-2

Variable (n:164) or (n/N)	Total
*Hb mg/dl	13.5 (1.9)
WBC count, × 10^9^/L	5800 (4400–7700)
Lymph count, × 10^9^/L	1250 (933–1670)
Platelet count, × 10^9^/L	191 (135–247)
ESR (124/163)	39.7 (11.0–74.0)
CRP mg/dl (159/163)	46 (30.2–65.7)
LDH, U/L (104/163)	472 (362.2–591.5)
AST, U/L (80/163)	39 (30.0–48.0)
ALT, U/L (80/163)	33 (24.0–41.0)
CPK U/L (23/163)	159 (51.0–236.0)
Sodium mmol/L (130/163)	136 (133–138)
Potassium mmol/L (130/163)	4.1 (3.8–4.4)
Magnesium mmol/L (60/163)	2.0 (1.9–2.2)
BUN mg/dl (158/163)	12.1 (9.0–16.7)
Cr mmol/L (158/164)	1.0 (0.9–1.2)

*Hb* hemoglobin, *WBC* white blood cell, *Lymph* lymphocyte, *ESR* erythrocyte sedimentation rate. *CRP* C-Reactive Protein, *LDH* lactate dehydrogenase, *AST* aspartate aminotransferase, *ALT* alanine aminotransferase, *CPK* creatine phosphokinase, *BUN* blood urea nitrogen, *Cr* creatinine

Positive CRP 85% (128/159) was our most common laboratory finding. High LDH 94.2% (98/104) and lymphocytic counts < 1100 41.4% (67/162) were other common laboratory data.

All patients were categorized into severe and non-severe groups in two ways. According to National Guideline [14], the first classification was done, which was in agreement with WHO recommendations, and the second one was CURB-65 criteria that are generally used for community-acquired pneumonia. According to National Guideline, the most common symptoms of disease onset and comorbidity in the severe group were nonproductive cough (69.7%), dyspnea (61.3%) and diabetes (23.5%), HTN (21.8%), respectively. Also, among vital signs and symptoms, mean O2 and nausea frequency showed a significant difference between the two groups (*P* < 0.05), but no significant difference was seen among comorbidities (Tables 4, 5). The laboratory findings and comparison among severity groups based on the National guideline and CURB-65 classification are noted in (Tables 6, 7).

**Table 4:** Clinical characteristics of patients based on severity groups, a national guideline

Variables	Non severe (n = 44)	Severe (n = 119)	*P* value
Age. Mean (SD)	51.6 (15.7)	54.8 (14.8)	0.573
Initial vital sign Mean (SD)			
RR breaths/min	18.4 (2.2)	19.5 (3.0)	0.174
Temp	37.0 (0.8)	37.1 (0.9)	0.574
PR	91.2 (13.2)	102.0 (20.3)	0.012
SBP	130.7 (18.2)	129.8 (20.9)	0.588
DBP	78.6 (10.5)	79.4 (13.5)	0.230
O2 SAT	93.2 (2.1)	90.2 (7.3)	< 0.001
Symptoms. no (%)			
Cough	34 (77.3)	83 (69.7)	0.343
Fever	22 (50.0)	61 (51.3)	0.886
Chill	14 (31.8)	46 (38.7)	0.422
Dyspnea	20.0 (45.5)	73.0 (61.3)	0.069
Sore throat	11 (25.0)	19 (16.0)	0.186
Headache	15 (34.1)	36 (30.3)	0.639
Dizziness	10 (22.7)	27 (22.7)	0.996
Weakness	22 (50.0)	58 (48.7)	0.886
Muscular pain	22 (50.0)	53 (44.5)	0.535
Diarrhea	11 (25.0)	16 (13.4)	0.078
Abdominal pain	5 (11.4)	9 (7.6)	0.442
Anorexia	22 (50.0)	51 (42.9)	0.416
Nausea	20 (45.5)	32 (26.9)	0.024
Vomiting	12 (27.3)	18 (15.1)	0.076
Date of symptoms before admission	7.0 (3.2–8.0)	6.0 (4.0–9.0)	0.835
Comorbidities. no (%)			
Diabetes	10 (22.7)	28 (23.5)	0.914
Hyperlipidemia	2 (4.5)	8 (6.7)	0.607
Hypertension	12 (27.3)	26 (21.8)	0.467
Chronic heart disease	7 (15.9)	17 (14.3)	0.795
Chronic kidney disease	0 (0.0)	1 (0.9)	0.537
Asthma	1 (2.3)	10 (8.4)	0.166
COPD	0 (0.0)	0 (0.0)	–
Cirrhosis	0 (0.0)	0 (0.0)	–
Autoimmune disease	0 (0.0)	1 (0.8)	0.542
History of malignancy	0 (0.0)	1 (0.8)	0.542
Recent chemoradiotherapy	0 (0.0)	1 (0.8)	0.542
Current steroid use	0 (0.0)	1 (0.8)	0.542
Immunosuppressant drug use	0 (0.0)	1 (0.8)	0.542

*RR* respiratory rate, *Temp* temperature, *PR* pulse rate, *Sys BP* systolic blood pressure, *Dias BP* diastolic blood pressure, *O2 Sat* O2 saturation, *CHD* Chronic heart disease, *CKD* chronic kidney disease, *COPD* chronic obstructive pulmonary disease

**Table 5:** Frequency of specific clinical and laboratory findings based on severity groups, a national guideline

Specific variables and lab findings n/N (%)	Non Severe (n = 44), No (%)	Severe (n = 119), No (%)	*P* value
RR > 24	0 (0)	9 (7.6)	0.061
Temp > 37.8	10 (22.7)	28 (23.5)	0.914
PR > 125	0 (0.0)	17 (14.3)	0.008
So2 < 90%	1 (2.3)	36 (30.3)	< 0.001
Fully Conscious patients	44 (100.0)	117 (98)	0.387
Non fully conscious patients	0 (0.0)	2 (2.0)	0.384
CRP > 6	30 (71.4)	98 (83.8)	0.084
CRP > 100	0 (0%)	15 (12.8)	0.015
PCR positive	32 (72.7)	79 (66.4)	0.441
PCR negative	12 (27.3)	40 (33.6)	0.441
Lymph < 1100	15 (34.1)	52 (44.1)	0.251
LDH > 245	0 (0.0)	98 (98)	< 0.001

*RR* respiratory rate, *Temp* temperature, *PR* pulse rate, *O2 Sat* O2 saturation, *CRP* C reactive protein, *PCR* polymerase chain reaction, *Lymph* lymph count, *LDH* lactate dehydrogenase

^*^This data in Median (IQR)

**Table 6:** Initial laboratory findings of patients infected with SARS-CoV-2 categorized according to national guidelines

Variable (N = 163) or (n/N)	Non severe	Severe	*P* value
*Hb mg/dl	13.7 (2.3)	13.4 (1.8)	0.122
WBC count, × 10^9^/L	5.6 (4.0–7.4)	5.8 (4.4–7.7)	0.573
Lymph count, × 10^9^/L	1.3 (0.9–1.7)	1.2 (0.8–1.6)	0.176
Platelet count, × 10^9^/L	196 (101–247.7)	194 (144.5–248)	0.866
ESR (124/163)	30.2 (3.8–63.1)	42.0 (13.0–81.6)	0.065
CRP mg/dl (159/163)	39.0 (22.0–69.0)	49.0 (32.0–65.0)	0.245
LDH, U/L (104/163)	240.5 (231.0–243.2)	476.0 (380.3–595.7)	0.001
AST, U/L (80/163)	36.0 (30.0–49.0)	40.0 (30–48.0)	0.555
ALT, U/L (80/163)	34.0 (22.0–41.0)	33.0 (24.5–42.0)	0.738
CPK U/L (23/163)	105 (27.8–182.2)	195.0 (58.2–311.0)	0.218
Sodium mmol/L (130/163)	136 (133–138)	136 (133–138)	0.820
Potassium mmol/L (130/163)	4.0 (3.7–4.3)	4.1 (3.9–4.5)	0.390
Magnesium mmol/L (60/163)	2.0 (1.9–2.3)	2.1 (1.9–2.3)	0.715
BUN mg/dl (158/163)	12.0 (0.9–15.4)	12.2 (0.9–17.5)	0.768
Cr mmol/L (158/164)	1.0 (0.9–1.2)	1.0 (0.9–1.2)	0.277

*Hb* hemoglobin, *WBC* white blood cell, *Lymph* lymphocyte, *ESR* erythrocyte sedimentation rate, *CRP*  C-reactive protein, *LDH* lactate dehydrogenase, *AST* aspartate aminotransferase, *ALT* alanine aminotransferase, *CPK* creatine phosphokinase, *BUN* blood urea nitrogen, *Cr* creatinine

^*^This data is Mean (SD), other data are Median (IQR)

**Table 7:** Initial laboratory findings of patients infected with SARS-CoV-2 categorized according to CURB-65

Variable (N = 158) or (n/N)	CURB-65 score ≤ 1	CURB-65 score ≥ 2	*P* value
*Hb mg/dl	13.8 (1.8)	12.4 (2.3)	< 0.001
WBC count, × 10^9^/L	5.6 (4.4–7.6)	5.3 (4.1–8.1)	0.343
Lymph count, × 10^9^/L	1.2 (0.9–1.6)	0.9 (0.7–0.7)	0.056
Platelet count, × 10^9^/L	189.0 (126.5–245.0)	214.5 (148.0–266.0)	0.121
ESR(124/158)	43.5 (25.7–58.5)	65.0 (48.7–98.0)	0.002
CRP mg/dl	37.0 (8.7–72.3)	62.6 (33.4–85.5)	0.020
LDH, U/L (104/158)	474 (365–600)	499.5 (349.5–586.5)	0.949
AST, U/L (80/158)	39.0 (31.0–48.0)	31.5 (24.5–48.5)	0.249
ALT, U/L (80/158)	34.0 (26.0–42.0)	27.0 (12.7–33.7)	0.026
CPK U/L (23/158)	121 (48–225)	190 (45.2–625)	0.990
Sodium mmol/L (130/158)	136 (133–138)	135 (131–136)	0.040
Potassium mmol/L (130/158)	4.1 (3.8–4.3)	4.0 (3.8–4.6)	0.790
Magnesium mmol/L (60/158)	2.0 (1.9–2.4)	2.0 (1.9–2.1)	0.444
BUN mg/dl	11.2 (8.8–14.5)	22.4 (17.6–26.2)	< 0.001
Cr mmol/L	1.0 (0.9–1.1)	1.1 (0.9–1.5)	< 0.001

*Hb* hemoglobin, *WBC* white blood cell, *Lymph* lymphocyte, *ESR* erythrocyte sedimentation rate, *CRP* C-reactive protein, *LDH* lactate dehydrogenase, *AST* aspartate aminotransferase, *ALT* alanine aminotransferase, *CPK* creatine phosphokinase, *BUN* blood urea nitrogen, *Cr* creatinine

^*^This data is Mean (SD), other data are Median (IQR)

Based on the CURB-65 classification, the most common symptoms of disease onset and comorbidity in the severe group were nonproductive cough (57.1%), dyspnea (57.1%), and HTN (46.4%), diabetes (42.9%), respectively. Besides, among vital signs and comorbidities, mean O2 saturation and diabetes, HTN, hyperlipidemia, chronic heart disease, and asthma showed a significant difference between the two groups (*P* < 0.05); however, no significant difference was observed in symptoms (Table 8, 9).

**Table 8:** Baseline characteristics of COVID-19 patients based on CURB-65 classification

Variables	CURB-65 score ≤ 1 (n = 130)	CURB-65 score ≥ 2 (n = 28)	*P* value
Age. Mean (SD)	50.3 (13.5)	70.1 (12.4)	< 0.001
Initial vital sign. Mean (SD)			
RR	19.1 (2.8)	19.3 (2.8)	0.785
Temp	37.0 (0.8)	37.3 (1.1)	0.060
PR	100.0 (17.7)	95.3 (25.7)	0.247
Sys BP	129.0 (20.2)	134.2 (20.1)	0.218
Dias BP	79.8 (11.8)	76.7 (16.2)	0.255
O2 SAT	91.9 (5.3)	87.6 (9.3)	< 0.001
Symptoms. no (%)			
Cough	96 (73.8)	16 (57.1)	0.078
Fever	67 (51.5)	13 (46.4)	0.624
Chill	50 (38.5)	9 (32.1)	0.531
Dyspnea	73 (56.2)	16 (57.1)	0.924
Sore throat	28 (21.5)	2 (7.1)	0.078
Headache	44 (33.8)	5 (17.9)	0.097
Dizziness	33 (25.4)	3 (10.7)	0.093
Weakness	63 (45.8)	15 (53.6)	0.624
Muscular pain	57 (43.8)	15 (53.6)	0.349
Diarrhea	23 (17.7)	3 (10.7)	0.366
Abdominal pain	13 (10.0)	1 (3.6)	0.278
Anorexia	61 (46.9)	11 (39.3)	0.462
Nausea	45 (34.6)	6 (21.4)	0.176
Vomiting	25 (19.2)	4 (14.3)	0.540
Comorbidities. no (%)			
Diabetes	23 (17.7)	12 (42.9)	0.004
Hyperlipidemia	6 (4.6)	4 (14.3)	0.057
Hypertension	24 (18.5)	13 (46.4)	0.002
Chronic heart disease	14 (10.8)	10 (35.7)	< 0.001
Chronic kidney disease	0 (0.0)	1 (0.9)	0.501
Asthma	5 (3.8)	4 (14.3)	0.031
COPD	0 (0.0)	0 (0.0)	–
Cirrhosis	0 (0.0)	0 (0.0)	–
Autoimmune disease	1 (0.8)	0 (0.0)	0.642
History of malignancy	1 (0.8)	0 (0.0)	0.642
Recent chemoradiotherapy	1 (0.8)	0 (0.0)	0.642
Current steroid use	1 (0.8)	0 (0.0)	0.642
Immunosuppressant drug use	1 (0.8)	0 (0.0)	0.642

*RR* respiratory rate, *Temp* temperature, *PR* pulse rate, *Sys BP* systolic blood pressure, *Dias BP* diastolic blood pressure, *O2 Sat* O2 saturation, *CHD* chronic heart disease, *CKD* chronic kidney disease, *COPD* chronic obstructive pulmonary disease

**Table 9:** Frequency of Specific clinical and laboratory findings based on CURB-65 classification

Specific variables and lab findings n/N (%)	CURB-65 score ≤ 1, (n = 130), No (%)	CURB-65 score ≥ 2, (n = 28) No (%)	P Value
RR > 24	7 (5.4)	1 (3.6)	0.691
Temp > 37.8	26 (20.0)	10 (35.7)	0.072
RR > 125	14 (10.8)	3 (10.7)	0.993
O2 Sat < 90%	23 (17.7)	12 (42.9)	0.004
Fully conscious patients	130 (100.0)	26 (92.9)	0.002
Non fully conscious patients	0 (0.0)	2 (7.0)	< 0.001
CRP > 6	98 (77.2)	25 (96.2)	0.026
CRP > 100	10 (7.9)	4 (15.4)	0.226
PCR positive	92 (70.8)	17 (60.7)	0.297
PCR negative	38 (29.2)	11 (39.3)	0.297
Lymph < 1100	46 (35.7)	20 (71.4)	< 0.001
LDH > 245	77 (95.1)	18 (90.0)	0.391

*RR* respiratory rate, *Temp* temperature, *PR* pulse rate, *O2 Sat* O2 saturation, *CRP* C-reactive protein, *PCR* polymerase chain reaction, *Lymph* lymphocyte count, *LDH* lactate dehydrogenase

^*^This data is Median (IQR)

Chi-square test for compatibility of severity between National guideline and CURB-65 showed that if a patient is non-severe according to National guideline, there is an 88% probability to be also in the non-severe group in CURB-65; otherwise, there is 19.3 compatibility in severe groups of National guideline and CURB-65.

## Discussion

Early diagnosis of 2019 novel coronavirus disease (COVID-19) is crucial for treating and controlling the disease. Compared to rRT-PCR, chest CT imaging may be a more reliable, practical, and rapid method to diagnose and assess COVID-19, especially in an epidemic area [13].

There was no significant difference in rRT-PCR results between severe and non-severe patients in our severity categories, namely CURB-65 and Iran National guideline for the diagnosis and the treatment of COVID-19 among outpatients and inpatients. No correlation was found between rRT-PCR results and symptom onset days before admission among patients or severity groups.

In our study, the nonproductive cough was the most common clinical symptom of the patients on admission, with dyspnea and fever as the following common symptoms. Our cough data is similar to Mohammad Ali Ashraf et al., who indicated that fever is not a specific finding in COVID-19. However, the cough has been a consistent clinical symptom in COVID-19 [18]. In two retrospective studies in Wuhan and Beijing, the most common clinical manifestations were fever, cough, shortness of breath, and fever, cough, fatigue, respectively [7, 11]

In a cohort of 41 patients with laboratory-confirmed 2019-nCoV infection in Wuhan, China, the most frequent symptoms at the onset of disease included fever, cough, and myalgia or fatigue, which was not consistent with our study [19].

The most prevalent presenting symptoms for COVID-19 include fever, cough, and shortness of breath. Extrapulmonary symptoms may occur early in the disease course. Gastrointestinal (GI) symptoms, including anorexia, nausea, vomiting, abdominal pain, and diarrhea, may occur early at the onset of the disease but are rarely the sole presenting feature [20].

GI symptoms are associated with COVID-19 in less than 10% of patients. In studies outside of China, there have been higher estimates. In 47 studies, meta-analyses, including 10,890 unique patients, nausea/vomiting was reported as the most common GI symptoms [21].

A recent meta-analysis of 4243 patients from China suggested that approximately 17.6% of patients had no gastrointestinal symptoms, including 9.2% with pain, 12.5% with diarrhea, and 10.2% with nausea/vomiting [22].

The most frequent GI symptoms were anorexia, nausea, and vomiting, similar to the mentioned studies in our research.

The median interval between illness onset to hospitalization was 6.5 (4.0–8.7) days, compared to 4.5 days and 7.0 days (4·0–8·0) in the study of Tian S. et al. and Huang Ch. et al., respectively [19, 23]. Positive CRP was our most common laboratory finding, followed by high LDH and lymphocytic counts < 1100 were the next common laboratory data, respectively. In confirmation of our findings, a recent meta-analysis revealed that the most frequent laboratory abnormalities were lymphopenia (35–75% of cases), increased CRP levels (75–93% of cases), LDH (27–92% of cases), and ESR (up to 85% of cases) [24]. Diabetes and hypertension were equally the most common comorbidities that were detected in our study. Similar to our study, in a retrospective study of 174 hospitalized patients with COVID-19 infection in Wuhan, the most common underlying comorbidities were chronic diseases such as hypertension and diabetes [25]. A cohort reported by Huang C. et al. showed that 31% of patients had an underlying disease, including diabetes [eight (20%)], hypertension [six (15%)], and cardiovascular disease [six (15%)] [19].

We categorized our patients into severe and non-severe groups based on Iranian National guidelines and the CURB-65 classification method to see differences in the distribution of comorbidities and significant clinical characteristics and laboratory results between them.

According to the National guideline, the frequency of O2 saturation < 90% was the most prevalent finding among the clinical indices of severity, and LDH > 245 was the most common laboratory finding (Table 6).

## Conclusion

There were no significant differences between positive and negative PCR test results in severity groups, indicating that the PCR result (true or false) cannot be associated with the severity of patients' disease.

There was compatibility between non-severe groups in CURB-65 classification and severity groups based on Iranian national guidelines, but there was no significant compatibility between severe groups. It should be noted that the CURB-65 classification could miss some severe cases in COVID-19.

We suggest that assessing patients' outcomes in severity groups based on CURB-65 and WHO guidelines should be considered in further studies. Applying different available severity scoring systems like Apache and Sofa in further investigations may lead to a comprehensive way of categorizing patients for better treatment.

### Limitations

This study has some limitations. First, because it was not initially possible to perform a rapid rRT-PCR test, the physicians judged the patient's hospitalization based on the lung's CT scan's clinical symptoms and severity. Therefore, we had no patients with a negative CT scan and positive rRT-PCR. Second, incomplete medical records of a few patients due to the high number of admissions to the hospital emergency ward, insufficient number of physicians and nurses to complete the history, and the patient's inability to express their history were among the limitations of this study. Third, given the lack of a national protocol suitable for testing to maintain integrity because of the economic burden for the patient, laboratory tests were performed at the physician's discretion, and the patient's clinical condition, and not all laboratory tests were performed for all patients, including CRP, ESR, CPK, LDH, and liver function test. Our samples' number was lower for further estimation and conclusion and did not seem to be predictable.

## Data Availability

The datasets used and/or analyzed during the current study are available from the corresponding author on reasonable request.

## Abbreviations

COVID-19: Coronavirus disease 2019
SARS-CoV-2: Severe acute respiratory syndrome coronavirus2
CURB-65: Confusion, uremia, respiratory rate, BP, age ≥ 65 years
rRT-PCR: Real-time reverse transcription polymerase chain reaction
CT: Computed tomographic
WHO: World Health Organization
IQR: Interquartile range
